# Omphalothoracopagus: A Case Report of Conjoined Twins and the Impact of Pelvimetry

**DOI:** 10.7759/cureus.90568

**Published:** 2025-08-20

**Authors:** Victoria M Estevez, Jose Perez Gongora, Murat Ibatullin, Paul Cervone, Kamil Yusupov

**Affiliations:** 1 Neonatology, Lake Erie College of Osteopathic Medicine, Bradenton, USA; 2 Obstetrics and Gynecology, Lake Erie College of Osteopathic Medicine, Bradenton, USA; 3 Medical Imaging, Lake Erie College of Osteopathic Medicine, Bradenton, USA; 4 Ultrasound, Kazan State Medical University and Interregional Clinical Diagnostic Center, Kazan, RUS

**Keywords:** conjoined twins, fetal anomalies, omphalothoracopagus, pelvimetry, prenatal diagnose

## Abstract

Pelvimetry, a radiographic imaging technique, is essential in evaluating pelvic dimensions and planning delivery methods, particularly in complex cases such as conjoined twins. This case report details the diagnosis of omphalothoracopagus twins in a 23-year-old G1P0 patient (first pregnancy with no prior deliveries) at 20 weeks of gestation using advanced imaging techniques, including 3D ultrasound, pulsed wave Doppler, and maximum intensity projection. Early prenatal imaging identified shared anatomy, including a single liver and a six-chambered heart, aiding in assessing surgical feasibility. Despite the advanced diagnostic tools, the severe anomalies precluded surgical separation, and the pregnancy was terminated. This case underscores the importance of pelvimetry and advanced imaging in guiding clinical decision-making and improving maternal and fetal outcomes.

## Introduction

This article was previously presented as a poster at the Lake Erie College of Osteopathic Medicine Interprofessional Research Day on April 24, 2025, and at the 2025 FMA Poster Symposium on July 26, 2025.

Pelvimetry is a medical imaging technique used to measure the dimensions and shape of the pelvic cavity, typically through radiographic methods like X-rays, CT scans, or MRI. Pelvimetry is useful to assess the size of a woman’s pelvis to determine the best mode of delivery [1]. The pelvis is critical in the use of body weight transfer and balance [2]. Pelvic anatomy shifts during pregnancy to accommodate the growing fetus. Normally, dramatic changes to pelvic structure due to misalignment of the pelvic bones or laxity of the pelvic ligaments lead to pelvic-perineal pain, urine and fecal incontinence, or pelvic organ prolapse [3]. The use of imaging techniques to obtain pelvic measurements throughout the course of pregnancy may be useful in preventing complications during the birthing process. In uncomplicated pregnancies, pelvimetry has been a useful tool to create birth plans tailored to expecting mothers, encouraging a less medically traumatic birthing experience, and preventing infant and maternal mortality.

Pelvimetry was historically used in obstetrics to assess the pelvic dimensions for concerns like cephalopelvic disproportion (CPD), where the baby's head or body may be too large to pass through the birth canal [4]. However, its routine use in obstetrics has declined with the development of more advanced imaging techniques like 3D ultrasound and MRI [5]. Pelvimetry can still be used today to determine the best delivery mode in the case of conjoined twins (e.g., omphalothoracopagus), where detailed measurements can provide insight for planning a surgical separation and reconstruction, if possible [4].

Multifetal pregnancies are classified by zygosity into monozygotic (identical) and dizygotic (fraternal) twins, and by chorionicity and amnionicity, based on whether the fetuses share a placenta or an amniotic sac. Dichorionic-diamniotic pregnancies, which have separate placentas and separate amniotic sacs, encompass all dizygotic twins and approximately 25% of monozygotic twins. Monochorionic-diamniotic twins share a single placenta but have distinct amniotic sacs, whereas monochorionic-monoamniotic twins share both a placenta and an amniotic sac. The latter configuration occurs exclusively in monozygotic twins and is the arrangement associated with conjoined twins.

The medical term used to describe conjoined twins ends with the suffix “pagus,” derived from the Greek word “fixed.” There are currently five types of conjoined twins that have been described: thoracopagus (joined at the thorax), omphalopagus (joined at the anterior abdominal wall), craniopagus (joined at the cranium), syncephalus (joined twins with one head), and ischiopagus (joined at the buttocks) [6].

Omphalothoracopagus is an extremely rare form of conjoined twinning, characterized by the fusion of two individuals at the level of the umbilicus and chest [7]. These conjoined twins typically share vital organs, including the heart, liver, and gastrointestinal systems. In addition to their anatomical fusion, conjoined twins share a single chorion, placenta, and amniotic sac [8]. The incidence of conjoined twins is rare, ranging from 1.5 per 100,000 births to one in 500,000, more often in females (3:1) [8]. Due to the complex anatomy and shared physiological systems, surgical management of such cases presents challenges, and in many cases, surgical intervention is not possible [9].

## Case presentation

**Figure 1:**
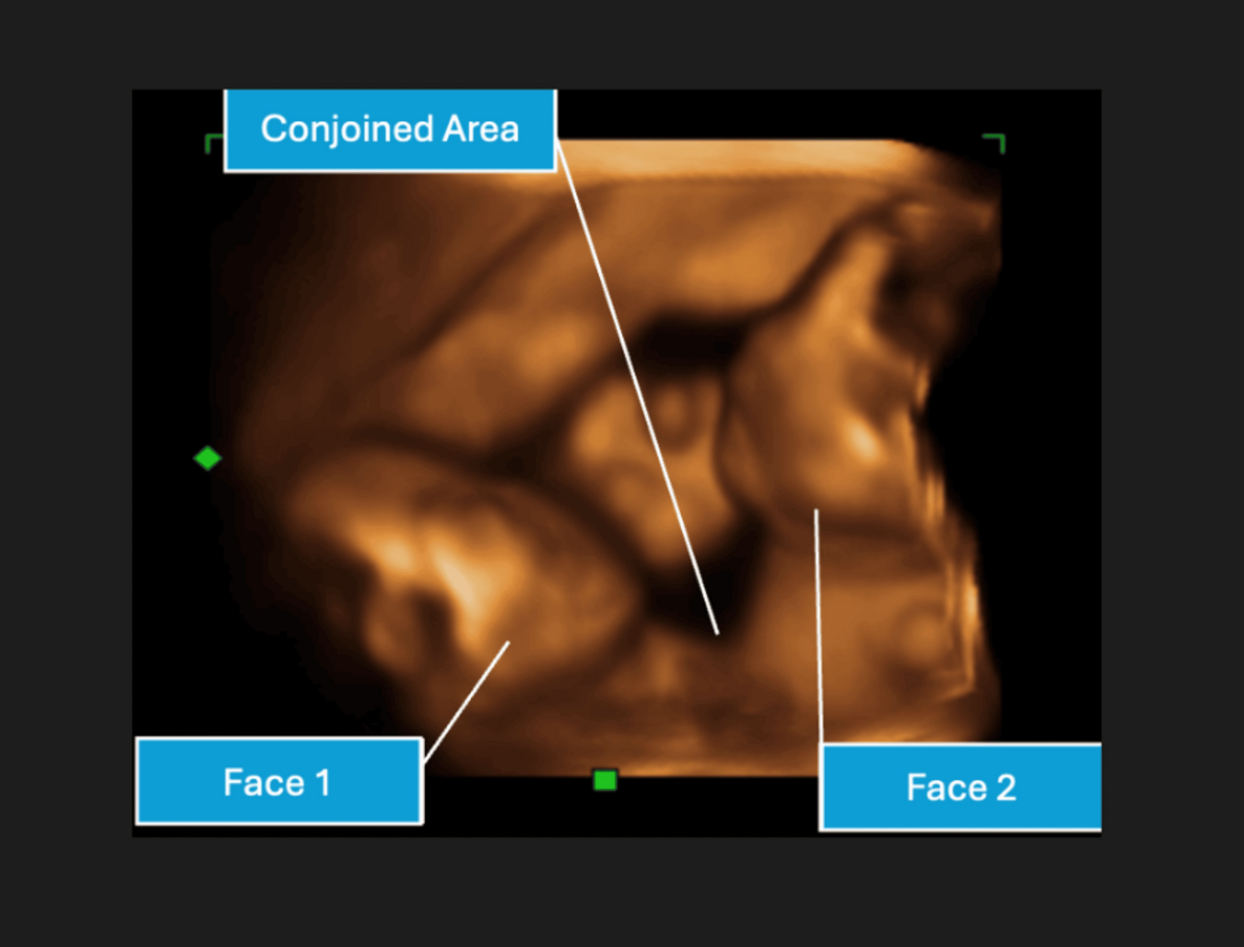
3D surface rendering mode showing a transverse view of 3D ultrasound. The blue labels depict the conjoined twins facing one another, with both faces labeled.

**Figure 2:**
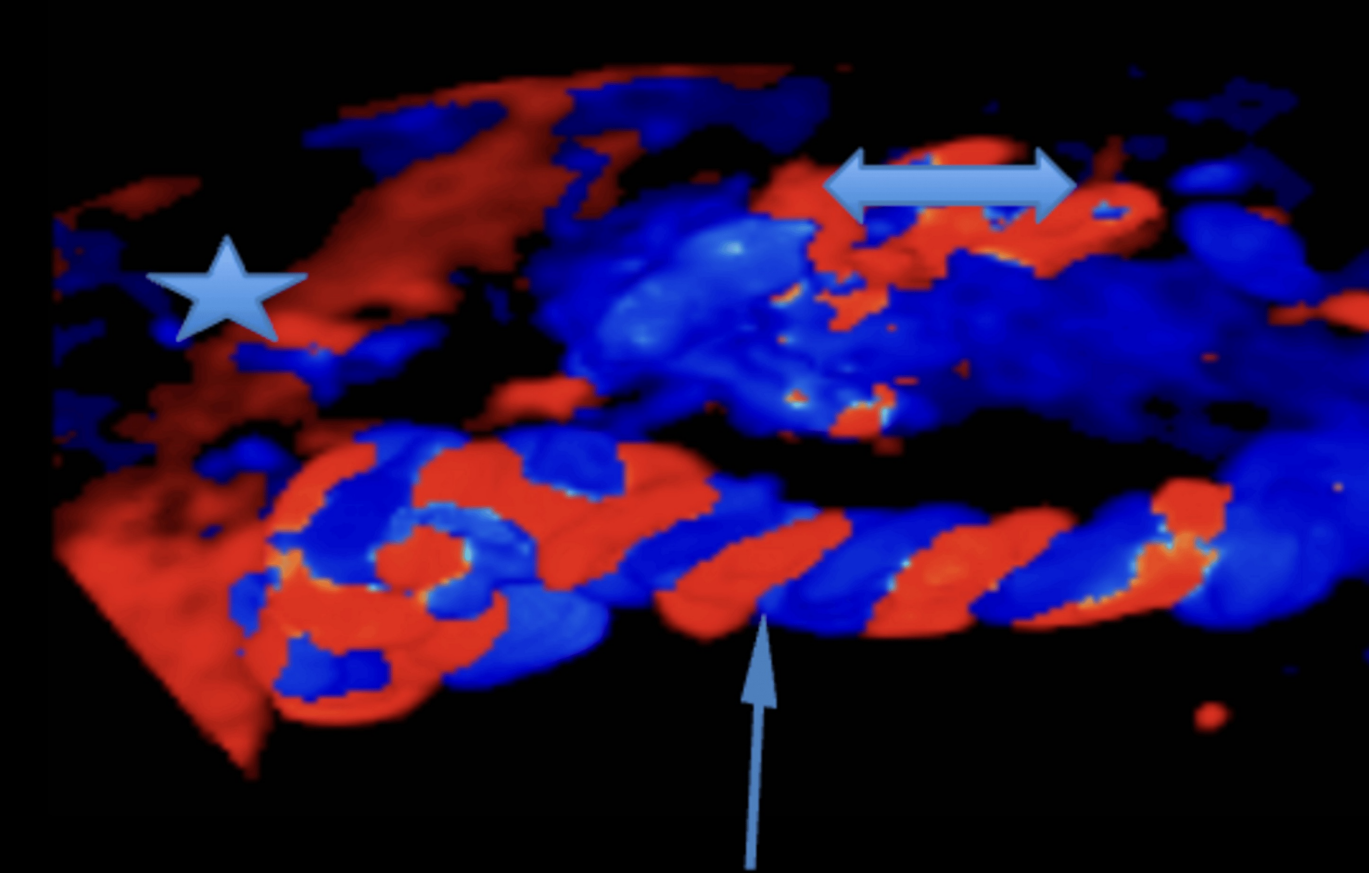
3D vascular mapping of umbilical circulation in omphalothoracopagus twins. This 3D reconstruction highlights the shared vascular anatomy in omphalothoracopagus conjoined twins. The arrow indicates a single umbilical cord, through which both twins receive a placental blood supply. The star marks the placenta, and the double-pointed arrow identifies the common fetal part, representing the region of anatomical fusion. Color Doppler delineates arterial (red) and venous (blue) flow, illustrating the complex vascular interdependence critical for preoperative planning. Note: Image spatial resolution is in millimeters (mm), with vessel measurements and anatomical distances interpreted at sub-centimeter accuracy.

The patient is a G1P0 (first pregnancy with no history of prior pregnancies) 23-year-old female admitted to the hospital on October 3rd, 2003, due to suspected undivided twins at 20 weeks of gestation to clarify the diagnosis and determine prognosis. The ultrasound examination was performed using a Voluson 730 ultrasound device with 3D/4D surface reconstruction techniques (Figure 1), 3D color flow mapping echocardiography (Figure 2), pulsed wave Doppler ultrasound, maximum intensity projection (MIP), multiplanar imaging, and tomographic imaging.

**Figure 3:**
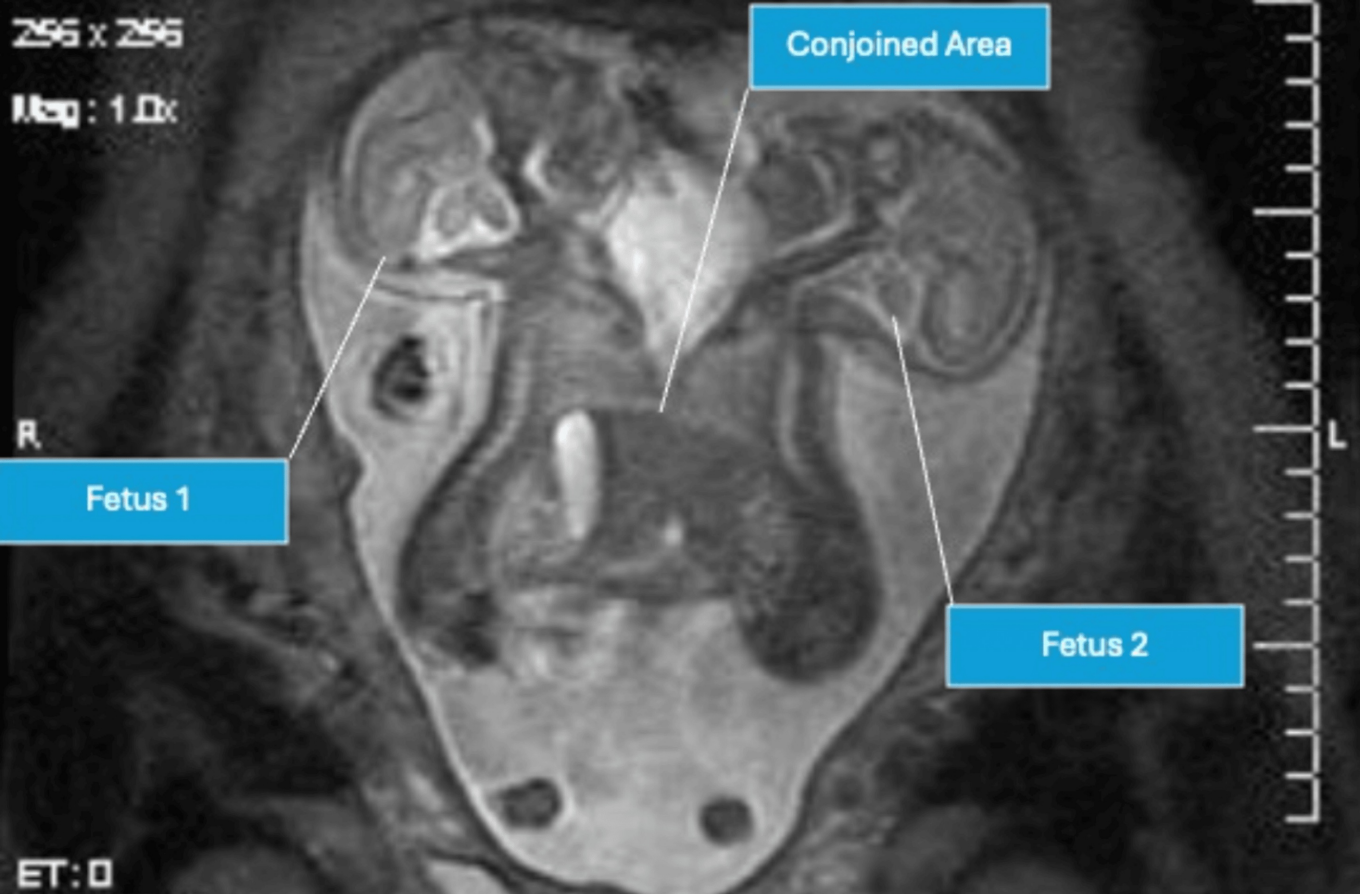
MRI image depicting the thoracic attachment of the conjoined twins, as indicated by the blue boxes.

MRI was performed in single-shot fast spin-echo (SSFSE), T1-weighted imaging (T1WI), and T2-weighted imaging (T2WI) modes, confirming the diagnosis of omphalothoracopagus twins (Figure 3). The malformed fetus shared a single liver and presented with a six-chambered heart and a common upper abdominal cavity.

The diagnosis was confirmed.

Due to the impossibility of surgical correction of the anomalies, the patient decided to terminate the pregnancy. The data from the morphological study after termination of pregnancy fully confirmed the findings of prenatal diagnosis. 

## Discussion

In cases of conjoined twins, a prenatal diagnosis can be made via ultrasound in the first trimester, as early as the 10th week of gestation [6]. To determine the exact type of conjoined twins, further imaging studies, such as 3D imaging, can help to determine where the twins are attached. As the pregnancy progresses, 3D reconstructive images are highly useful to provide insight into surgical management options [10,11]. In some cases, the use of 3D printed models allows even further evaluation of the twins to pre-surgically plan, unless termination is recommended by the physician. This planning reduces the chances of mortality, lowers costs, decreases operating room time, and improves patient outcomes [10]. Understanding the pelvic dimensions, as well as having an idea about the exact size of the twins and their vital organs, allows for improvement in outcomes [10].

3D sonography, as a complementary exam to conventional 2D imaging, offers a quick and non-invasive method for obtaining detailed images and visualizing the pelvis in cases of pelvimetry, previously achievable only through CT or MRI [10]. One key advantage of this technique is 3D multiplanar imaging, which enables visualization of a single section from three orthogonal planes (transverse, sagittal, and coronal) intersecting at right angles. This capability enhances the assessment of complex anatomy and provides valuable information about fetal organ volume, 3D color Doppler imaging, and surface anatomy. However, results may be affected by factors such as oligohydramnios and motion, which can degrade image quality [10].

The integration of pulsed wave Doppler and MIP in ultrasound imaging has significantly advanced the understanding of vascular structures. Pulsed wave Doppler provides essential data on blood flow dynamics, such as velocity and the presence of turbulence or stenosis, which are critical for diagnosing and evaluating vascular conditions [12]. In contrast, MIP enhances visualization by generating high-resolution images, enabling better assessment of anatomical details [12].

The combination of pulsed wave Doppler with 3D MIP imaging is particularly powerful. This synergy provides a more comprehensive view of the vasculature, allowing for a better understanding of complex vascular anatomy [12], including the identification of abnormalities or irregularities. This is invaluable in clinical applications such as surgical planning, especially in intricate procedures like the delivery and separation of conjoined twins, where precise visualization of blood vessels is critical to ensure that surgeries are safe and effective.

Some imaging techniques used in pelvimetry can also help identify congenital defects in fetuses that may not be directly related to the conjoined organs. Commonly detected defects include neural tube anomalies and orofacial clefts, which could further complicate clinical management and impact the long-term outcomes for the fetuses [13]. These additional findings can provide valuable insights into the potential quality of life and associated comorbidities, which could influence the feasibility and planning of separation procedures. Early prenatal assessment is essential for planning, as it helps determine the required multidisciplinary team, evaluate the morbidity and mortality associated with separation, and gather information to guide the family in making an informed decision [14].

A study published in the Egyptian Journal of Radiology and Nuclear Medicine examined the role of pelvimetry techniques, including 3D ultrasound and MRI, in the diagnosis and management of rare cephalopagus twins, particularly the non-janiceps type [15]. Cephalopagus twins are uncommon and classified into the janiceps type, characterized by two faces on opposite sides of the head, and the even rarer non-janiceps type, which presents with a single head and face. In the reported case, imaging confirmed the number of umbilical vessels and delineated the anatomical structures shared by the fetuses, but the pregnancy was ultimately terminated. The authors emphasized the value of antenatal MRI in such cases, as it provides superior resolution, reduced motion artifacts, and a more detailed assessment of fetal abnormalities compared with ultrasound. They also noted that, depending on the extent and location of fusion, highly conjoined twins can be challenging to diagnose accurately with ultrasound alone, sometimes resulting in an initial misdiagnosis of a singleton pregnancy, a pitfall particularly noted in cephalopagus cases. Research on pelvimetry in cephalopagus twins remains limited, and similar measurements in other subtypes of conjoined twins, such as thoracopagus and omphalopagus, have been reported only sporadically, suggesting a need for broader investigation to guide diagnosis and perinatal planning.

In terms of prognosis, outcomes for conjoined twins vary widely depending on the type and extent of fusion. A study involving 40 conjoined twin pregnancies found that thoracopagus was the most common type (72.5%), followed by omphalopagus (5%), with the remainder consisting of other fusion types [16]. Of these pregnancies, 58.8% were electively terminated. Among those that continued, the average gestational age at delivery was approximately 35 weeks. Unfortunately, the postnatal mortality rate was high, with 88% of newborns dying shortly after birth. Surgical separation, when attempted, resulted in a survival rate of 60% among live-born infants, translating to an overall survival rate of just 7.5%. These sobering statistics highlight the critical need for precise prenatal diagnosis and counseling to inform decision-making and management in these complex cases.

## Conclusions

Pelvimetry serves a pivotal role in assessing the anatomical configuration of conjoined twins. Through its ability to measure pelvic dimensions, evaluate the degree of fusion, and guide surgical planning, pelvimetry provides insights that have significant impacts on the chances of infant and maternal mortality. As the medical community continues to encounter these rare cases, pelvimetry approaches will continue to serve as a useful marker to avoid preventable complications.
